# Re-Expression of the Lorenz Asymmetry Coefficient on the Rotated and Right-Shifted Lorenz Curve of Leaf Area Distributions

**DOI:** 10.3390/plants14091345

**Published:** 2025-04-29

**Authors:** Yongxia Chen, Feixue Jiang, Christian Frølund Damgaard, Peijian Shi, Jacob Weiner

**Affiliations:** 1 Co-Innovation Center for Sustainable Forestry in Southern China, Bamboo Research Institute, College of Civil Engineering, Nanjing Forestry University, Nanjing 210037, China; yongxia@njfu.edu.cn (Y.C.); fxjiang@njfu.edu.cn (F.J.); 2 Department of Ecoscience Terrestrial Ecology, Aarhus University, 8000 Aarhus, Denmark; 3 Department of Plant and Environmental Sciences, University of Copenhagen, Thorvaldsensvej 40, 1871 Frederiksberg, Denmark; jw@plen.ku.dk

**Keywords:** asymmetry measures, leaf area distributions, Lorenz curve, performance equation, *Shibataea chinensis* Nakai

## Abstract

The Gini coefficient, while widely used to quantify inequality in biological size distributions, lacks the capacity to resolve directional asymmetry inherent in Lorenz curves, a critical limitation for understanding skewed resource allocation strategies. To address this, we extend our prior geometric framework of the rotated and right-shifted Lorenz curve (RRLC) by introducing two original asymmetry metrics: the positional shift ratio (*P_L_*, defined as $x_{c}/\sqrt{2}$, where $x_{c}$ is the *x*-coordinate of the RRLC’s maximum value point) and the area ratio (*P_A_*, defined as *A_L_*/(*A_L_* + *A_R_*), where *A_L_* and *A_R_* denote the areas under the left and right segments of the RRLC). These indices uniquely dissect contributions of dominant versus small individuals to overall inequality, with *P_L_* reflecting the peak position of the RRLC and *P_A_* quantifying the area dominance of its left segment. Theoretically, *P_L_* directly links to the classical Lorenz asymmetry coefficient *S* (defined as $S=x_{c}^{\prime }+y_{c}^{\prime }$, where $\left(x_{c}^{\prime },y_{c}^{\prime }\right)$ is the tangent point on the original Lorenz curve with a 45° slope) through *S* = 2 − 2*P_L_*, bridging geometric transformation and parametric asymmetry analysis. Applied to 480 *Shibataea chinensis* Nakai shoots, our analysis revealed that over 99% exhibited pronounced left-skewed distributions, where abundant large leaves drove the majority of leaf area inequality, challenging assumptions of symmetry in plant canopy resource allocation. The framework’s robustness was further validated by the strong correlation between *P_A_* and *P_L_*. By transforming abstract Lorenz curves into interpretable bell-shaped performance curves, this work provides a novel toolkit for analyzing asymmetric size distributions in ecology. The proposed metrics can be applied to refine light-use models, monitor phenotypic plasticity under environmental stress, and scale trait variations across biological hierarchies, thereby advancing both theoretical and applied research in plant ecology.

## 1. Introduction

Plant leaves are the primary sites of photosynthesis, serving as the biochemical factories that convert light energy, carbon dioxide, and water into organic compounds essential for growth and survival. As the principal interface between plants and their environment, leaves exhibit remarkable plasticity in morphology and physiology, enabling adaptation to heterogeneous light conditions, which is a critical factor influencing photosynthetic efficiency and plant fitness [1,2]. Given their central role in carbon assimilation, understanding leaf traits and their spatial distribution within plants is important in deciphering ecological strategies and resource utilization. In broad-leaved plants, variation in leaf area allocation across individual shoots or entire canopies reflects adaptive responses to light heterogeneity [3,4]. A well-documented phenomenon is the differentiation between sun and shade leaves. Sun leaves, typically exposed to high irradiance, are characterized by smaller areas, thicker laminas, higher stomatal density, and greater photosynthetic capacity per unit area. These traits enhance water-use efficiency and reduce photodamage under intense light. In contrast, shade leaves, situated in dimmer understory or lower canopy layers, exhibit larger areas, thinner tissues, and lower mass per unit area, maximizing light capture in low-light environments [5,6,7,8]. This intra-canopy plasticity in leaf morphology enables plants to optimize light interception across vertical gradients. For instance, in tree crowns, upper canopy leaves prioritize high-light adaptation, while lower leaves prioritize shade tolerance, thereby collectively enhancing whole-plant carbon gain [9]. Similarly, within a single shoot, leaf area distribution often follows a basipetal gradient, with younger apical leaves smaller and thicker and older basal leaves larger and thinner, aligning with age-dependent light exposure [10,11]. Such patterns suggest that plants strategically allocate leaf area to balance energy investment with photosynthetic returns under spatially variable light. These adaptations are further modulated by species-specific trade-offs between growth rate and stress tolerance, as seen in the contrasting strategies of pioneer versus shade-tolerant species [12]. Collectively, these studies underscore that leaf area distribution within shoots or individuals is not merely a passive outcome of development but a dynamic process driven by selective pressures to maximize light utilization efficiency. In this context it is useful to have a practical method to quantify the inequality of leaf area distributions per shoot or per plant and examine which leaves have contributed to the inequality. This is helpful to account for the strategies for optimizing the use of light by plants.

**Figure 1:**
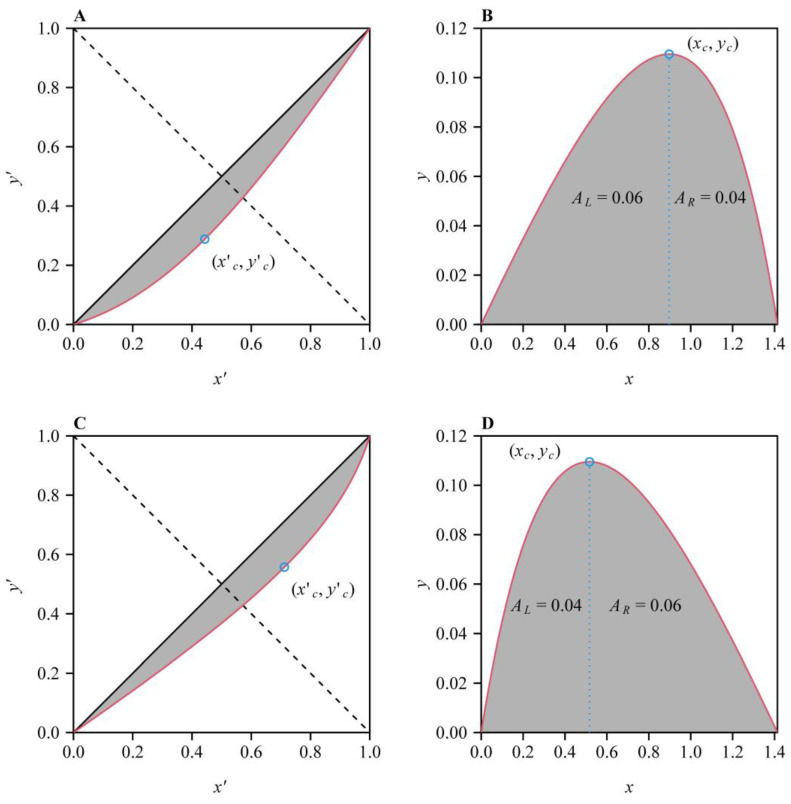
The Lorenz curves (**A**,**C**) and their rotated and right-shifted forms (**B**,**D**). $\left(x_{c}^{\prime },y_{c}^{\prime }\right)$ (the blue open circle) in panels (**A**,**C**) is the point of tangency at which the tangent of the Lorenz curve is parallel to the egalitarian line through the points (0, 0) and (1, 1). The dashed line through the two points (0, 1) and (1, 0) is defined as the axis of symmetry. Panels (**B**,**D**) exhibit the rotated and right-shifted Lorenz curves, which can be described by the performance equation. $\left(x_{c},\;y_{c}\right)$ is the maximum value point of the performance curve, which is obtained by rotating $\left(x_{c}^{\prime },y_{c}^{\prime }\right)$ counterclockwise by 3/4π and shifting it to the right by $\sqrt{2}$. In panels (**B**,**D**), *A_L_* represents the area of the region formed by the performance’s left part and the *x*-axis, and *A_R_* represents the area of the region formed by the performance’s right part and the *x*-axis.

The Lorenz curve was originally developed to evaluate inequality in income distributions by plotting the cumulative percentage of the sorted household income in ascending order (*y*′) against the cumulative percentage of the number of households in an economy (*x*′) (Figure 1A,C) [13,14,15]. The Gini coefficient [16], which is a measure of inequality, is defined as the proportion of the area formed by the Lorenz curve and the line of absolute equality (i.e., the line segment from (0, 0) to (1, 1)) to the area formed by the line of absolute equality and the two coordinate axes (Figure 1A,C). The Lorenz curve and the Gini coefficient have also been used to measure the inequality of biological size distributions [17], such as the distributions of fruit volumes in a vine, leaf areas in a shoot, stomatal areas in a micrograph, petal areas in a flower, and tree diameters at breast height in a quadrat [18]. The Gini coefficient is a summary statistic that does not capture all the information present in the Lorenz curve [19]. Furthermore, multiple distinct Lorenz curves can share the same Gini coefficient. The Lorenz curve reveals three size distribution patterns: (i) inequality driven by abundant large individuals, (ii) inequality dominated by a few large individuals, and (iii) parity in contributions between small and large individuals. If the Lorenz curve is a continuous function, there is a point on the Lorenz curve (denoted as **the point of tangency**, i.e., $\left(x_{c}^{\prime },y_{c}^{\prime }\right)$ in Figure 1A,C) where the derivative equals tan(π/4), i.e., the tangent of the Lorenz curve at this point is parallel to the egalitarian line. For pattern (i), the point of tangency is located in the lower left area relative to the straight line through (1, 0) and (0, 1), denoted as **the axis of symmetry** (Figure 1A); for pattern (ii), the point of tangency is located in the upper right area relative to the axis of symmetry (Figure 1C); for pattern (iii), the point of tangency is exactly the intersection point between the Lorenz curve and the axis of symmetry. It is apparent that pattern (iii) signifies a symmetrical Lorenz curve about the axis of symmetry.

Let *x*′ represent the cumulative percentage of the number of individuals in a statistical unit (e.g., the percentage of the number of fruits in a vine, that of stomata in a micrograph, that of leaves in a shoot, that of tree diameters at breast height in a quadrat), and *y*′ represent the cumulative percentage of size, i.e., the horizontal and vertical coordinates of the Lorenz curve (Figure 1A,C). Let *x*′ + *y*′ = *C* ⟺ *y*′ = −*x*′ + *C*, where *C* is a constant between 0 and $\sqrt{2}$. The equation can generate a group of *y*′ vs. *x*′ straight lines with the same slope being equal to negative unity in the *x*′−*y*′ plane. Let $S=x_{c}^{\prime }+y_{c}^{\prime }$, where $x_{c}^{\prime }$ and $y_{c}^{\prime }$ are the horizontal and vertical coordinates of the point of tangency [19]. It is apparent that *S* < 1 indicates pattern (i) for overall inequality; *S* > 1 indicates pattern (ii) for overall inequality; *S* = 1 indicates pattern (iii). Our prior study shows that after the Lorenz curve of biological size distributions is rotated counterclockwise by 3/4π and then shifted to the right by $\sqrt{2}$, it becomes a skewed or symmetrical bell-shaped curve (Figure 1B,D) that can be well described by a nonlinear equation called the performance equation [11,18]. The performance equation was first proposed to describe the effect of body temperature (*x*) on the jumping distance (*y*) of green frogs [20], which has its original form written as follows:

(1) $$y=c\left(1-e^{-K_{1}\left(x-x_{1}\right)}\right)\left(1-e^{K_{2}(x-x_{2})}\right),$$

where *c*, *K*_1_, *K*_2_, *x*_1_, and *x*_2_ are the parameters that can be estimated by minimizing a target function, e.g., the residual sum of squares (RSS) between the observed and predicted *y* values, using the nonlinear least-squares method [21,22,23]. In the present study, *x* and *y* represent the horizontal and vertical coordinates the rotated and right-shifted Lorenz curve (denoted as RRLC) to replace their original meanings of body temperature and jumping distance; the original temperature axis (i.e., the *x*-axis) then represents the rotated and right-shifted egalitarian line, and *x*_1_ and *x*_2_ exactly correspond to its two endpoints, i.e., (0, 0) and ($\sqrt{2}$, 0) (Figure 1B,D). Thus, Equation (1) can be simplified to the following form [11]:

(2) $$y=c\left(1-e^{-K_{1}x}\right)\left(1-e^{K_{2}(x-\sqrt{2})}\right).$$

Here, *c* is a scaling parameter influencing the maximum value of the performance curve (i.e., the curve generated by the performance equation representing the RRLC); *K*_1_ and *K*_2_ determine the curvature of the performance curve’s left part and that of the performance curve’s right part about the vertical line through the maximum value point on the performance curve. Since *x*_1_ and *x*_2_ are predefined model parameters (i.e., the endpoints of the RRLC) in Equation (1), only the parameters *c*, *K*_1_, and *K*_2_ in Equation (2) require estimation once experimental data are available. When *K*_1_ is equal to *K*_2_, the performance curve is bilaterally symmetrical about $x=\sqrt{2}/2$. And the point of tangency in the *x*′-*y*′ plane corresponds to the maximum point on the RRLC in the *x*–*y* plane (Figure 1). To increase the flexibility of data fitting of the performance equation, ref. [11] also proposed a generalized version of the performance equation, written as follows:

(3) $$y=c{\left(1-e^{-K_{1}x}\right)}^{a}{\left(1-e^{K_{2}(x-\sqrt{2})}\right)}^{b},$$

where *a* and *b* are two additional parameters that also can be estimated by minimizing the RSS between the observed and predicted *y* values using the nonlinear least-squares method [21,22,23]. The performance equation and its generalized version were validated to fit the rotated and right-shifted observations of *y*′ vs. *x*′ in biological size distributions regardless of the size distribution function [18,24].

It is easy to determine whether the Lorenz curve is asymmetrical or symmetrical by observing the shape of the performance curve in the *x–y* plane. Let $\left(x_{c},\;y_{c}\right)$ represent the rotated and right-shifted point of tangency, which is the maximum value point on the RRLC (Figure 1B,D). To determine the degree of asymmetry of the RRLC, we only need to examine the numerical value of $x_{c}/\sqrt{2}$ (denoted as *P_L_*) or the proportion of the area of the region formed the left part of the performance curve and the *x*-axis to the whole area of the region formed by the performance curve and the *x*-axis (denoted as *P_A_*). If *P_L_* > 0.5 or *P_A_* > 0.5, the RRLC is left-skewed; if *P_L_* < 0.5 or *P_A_* < 0.5, the RRLC is right-skewed; if *P_L_* = 0.5 or *P_A_* = 0.5, the RRLC is bilaterally symmetrical about the vertical line *x* = *x_c_*. In the present study, we examined the relationships among the two asymmetry measures for the RRLC in the *x*–*y* plane here and the Lorenz asymmetry coefficient (*S*) in the *x*′*-y*′ plane using 480 shoots of *Shibataea chinensis* Nakai, an herbaceous bamboo species. This work can provide a useful tool for detecting the contribution of the largest leaves to the inequality of leaf area distribution per shoot or per individual plant.

While the Gini coefficient has been widely adopted to quantify inequality in biological size distributions, its inherent limitation in capturing the directional asymmetry of Lorenz curves necessitates novel analytical frameworks. In this study, we introduce the RRLC coupled with two redefined asymmetry metrics (i.e., *P_L_* and *P_A_*). This is a methodological advancement that uniquely disentangles the contributions of dominant large individuals from cumulative small-individual effects. Unlike conventional approaches that rely solely on the Lorenz asymmetry coefficient (*S*), our RRLC-based metrics leverage geometric transformations and performance equation parameterization to provide dual perspectives on distributional skewness: *P_L_* quantifies positional shifts of the RRLC peak, while *P_A_* integrates area ratios between left and right segments of the performance curve. This dual-metric system addresses a critical gap in ecological inequality analysis by enabling multidimensional characterization of resource allocation strategies, particularly in light-limited environments where large leaves disproportionately drive photosynthetic efficiency. Beyond plant ecology, our framework offers a generalizable tool for analyzing asymmetric size distributions in fields ranging from canopy physiology to biodiversity conservation, where disentangling outlier-driven inequality is essential for mechanistic modeling.

## 2. Materials and Methods

### 2.1. Leaf Sampling

We randomly sampled 480 *S. chinensis* shoots growing in the Nanjing Forestry University Xinzhuang campus (118°48′53″ E, 32°4′52″ N) in the autumn of 2023. The number of leaves per shoot ranges from 8 to 40. The shoots were cut at the ground, then wrapped with wet paper, and taken back to the lab within one hour. All leaves were sampled from each of the 480 shoots, and the pseudo-petioles of leaves were removed.

### 2.2. Data Acquisition

All leaves on the 240 shoots were scanned at a 1:1 scale to .jpg images with a photo scanner (V550, Epson Indonesia, Batam, Indonesia) at a resolution of 600 dpi and then transformed into black-and-white .bmp images using Adobe Photoshop (version 9.0; Adobe, San Jose, CA, USA). An M-file in Matlab (version ≥ 2009a; MathWorks, Natick, MA, USA) presented in [25,26] was used to extract leaf boundary coordinates in the .bmp images. The 240 shoots comprised 4638 leaves in total, with each leaf containing 4809 ± 865 boundary points (mean ± SD) extracted from digitized images. To balance computational efficiency with geometric fidelity, a uniform subsample of 2000 boundary points for each leaf was selected to calculate the leaf area (*A*) using the “bilat” function in the “biogeom” package (v1.3.5) [27] implemented in R statistical software (v4.2.0) [28]. Here, the “bilat” function calculates polygon area by first identifying adjacent boundary points through minimum Euclidean distance analysis and then applying the shoelace formula to the ordered coordinate sequence, thereby reconstructing closed contours for precise areal computation. Due to the excessive scanning workload, we did not directly scan the leaves for the other 240 shoots to determine their *A* values. For all leaves of the other 240 shoots (5455 leaves in total), we measured the length and width of each leaf and calculated individual *A* using the Montgomery equation that assumes *A* to be proportional to the product of leaf length (*L*) and width (*W*) [29,30]:

(4) A=kLW,

where *k* is the proportionality coefficient to be estimated. It is equal to two-thirds, which was validated using the 240 individual leaves of this bamboo species [31].

The raw data of leaf area of the 240 *S. chinensis* shoots, and the raw data of leaf length and width of the other 240 *S. chinensis* shoots are accessible from the online Supplementary Tables S1 and S3 in [31].

### 2.3. Three Indicators for Measuring the Asymmetry of the Lorenz Curve

We used three measures for quantifying the degree of asymmetry of the Lorenz curve as mentioned above. The asymmetry Lorenz coefficient takes the following form as [19]:

(5) $$S=x_{c}^{\prime }+y_{c}^{\prime },$$

where $x_{c}^{\prime }$ and $y_{c}^{\prime }$ are the horizontal and vertical coordinates of the point of tangency in the *x*′–*y*′ plane (Figure 1A,C). When *S* < 1, the RRLC is left-skewed; when *S* > 1, the RRLC is right-skewed; when S = 1, the RRLC is bilaterally symmetrical about the vertical line *x* = *x_c_* (Figure 1B,D).

The proportion of the line segment from the point (0, 0) to the point (*x_c_*, 0) (i.e., the point on the *x*-axis associated with the maximum value of the performance curve) to $\sqrt{2}$ on the RRLC, which is denoted as *P_L_*:

(6) $$P_{L}=x_{c}/\sqrt{2}.$$

When *P_L_* > 0.5, the RRLC is left-skewed; when *P_L_* < 0.5, the RRLC is right-skewed; when *P_L_* = 0.5, the RRLC is bilaterally symmetrical about the vertical line *x* = *x_c_*. There is an explicit linear relationship between *S* and *P_L_*: *S* = 2 − 2*P_L_* (Appendix A).

The proportion of the area of the region formed by the performance curve’s left part (*A_L_*) and the *x*-axis to the area of the region formed by the whole performance curve (i.e., the RRLC) and the *x*-axis (i.e., the sum of the left part’s area and the right part’s area (*A_L_* + *A_R_*); Figure 1B,D), which is denoted as *P_A_*, written as follows:

(7) $$P_{A}=A_{L}/\left(A_{L}+A_{R}\right).$$

When *P_A_* > 0.5, the RRLC is left-skewed; when *P_A_* < 0.5, the RRLC is right-skewed; when *P_A_* = 0.5, the RRLC is bilaterally symmetrical about the vertical line *x* = *x_c_*. *P_A_* is similar to *P_L_*. However, *P_A_* contains more information, including the maximum value point of the performance curve.

Because *S* and *P_L_* have an explicit linear relationship (Appendix A), we used the reduced major axis (RMA) regression [32] to test the probable relationship between *P_L_* and *P_A_*. The RMA regression estimates the slope to be the square root of the quotient of the variance of the dependent variable and that of the independent variable if the covariance of the dependent and independent variables is positive. If we exchange the dependent and independent variables, the estimated slope is the reciprocal of the estimated slope of the unexchanged dependent and independent variables.

The first derivation of the performance equation was provided in [33]. However, there is no analytical solution for the maximum value point of the performance curve. We calculated the numerical solution of *x_c_* and *y_c_* (see below for details).

### 2.4. Parametric Estimation of the Performance Equation

We used Equation (2) to describe the rotated and right-shifted Lorenz curve (RRLC). The parameters of Equation (2) were estimated by minimizing the residual sum of squares (RSS) between the observed and predicted *y* values using the “L-BFGS-B” optimization algorithm [34]. The lower and upper bounds for the three parameters, i.e., *c*, *K*_1_ and *K*_2_, were set to be (0, $\sqrt{2}/2$), (0, 50), and (0, 50), respectively, in nonlinear regression. The root-mean-square error (RMSE = $\sqrt{RSS/n}$) is usually used to reflect the goodness of fit of nonlinear regression, where *n* represents the total number of leaves in a shoot. However, given the variation of the maximum value point on the RRLC across shoots, we used an adjusted root-mean-square error (RMSE_adj_), which equals the RMSE divided by the maximum value of the performance curve [35], written as follows:

(8) $${\mathrm{RMSE}}_{\mathrm{adj}}=\frac{\sqrt{\mathrm{RSS}/n}}{y_{c}}.$$

As a rule of thumb, a < 0.05 RMSE_adj_ indicates a good fit.

### 2.5. Calculation of the Gini Coefficient for the Leaf Area Distribution per Shoot

After obtaining the parameters of Equation (2), the Gini coefficient is calculated using the following formula [11]:

(9) $$\mathrm{Gini}\;\mathrm{coefficient}=2\times \int_{0}^{\sqrt{2}}y\left(x;\;\hat{\theta }\right)dx$$

where *y* is given by Equation (2), and $\hat{\theta }$ represents the parametric vector including the estimates of *c*, *K*_1_, and *K*_2_.

The calculations of the above four indices were carried out using the statistical software R (v4.2.0) [28]. The nonlinear regression was formed using the “optim” function.

## 3. Results

**Figure 2:**
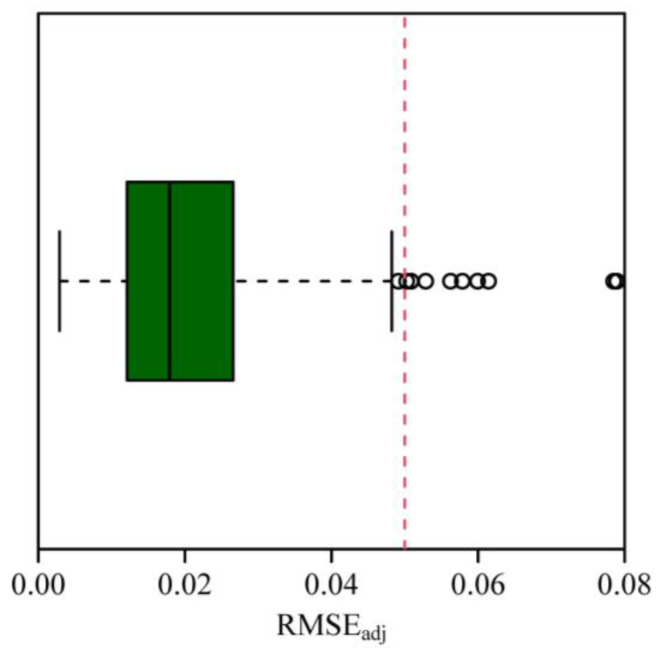
Box plot of the adjusted root-mean-square errors (Equation (8)) of the performance equation fit to the 480 shoots. Here, the vertical dashed line represents the value of 0.05.

**Figure 3:**
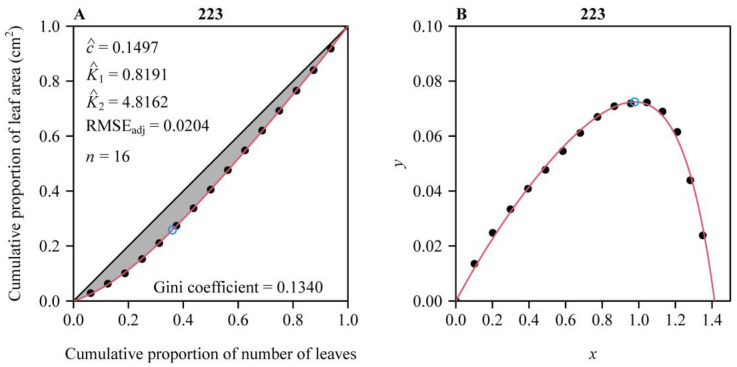
Fitted results for an individual shoot (the 223rd shoot) of *S. chinensis* using the performance equation. Panel (**A**) shows the comparison of the observations and the predicted original Lorenz curve, and the 45° straight line represents the egalitarian line; panel (**B**) shows the comparison of the observations and the predicted Lorenz curve after being rotated and right-shifted. The closed circles represent the observations, and the red curve represents predicted values; the open circle represents the point of tangency in panel (**A**), or the maximum value point of the performance curve in panel (**B**). In panel (**A**), the letters *c*, *K*_1,_ and *K*_2_ with hats represent the estimated parameters of the performance equation; RMSE_adj_ represents the adjusted root-mean-square error; and *n* represents the number of leaves on the individual shoot. The Gini coefficient is estimated as double the area of the region between the performance curve (i.e., the red curve in panel (**B**)) and the *x*-axis.

**Figure 4:**
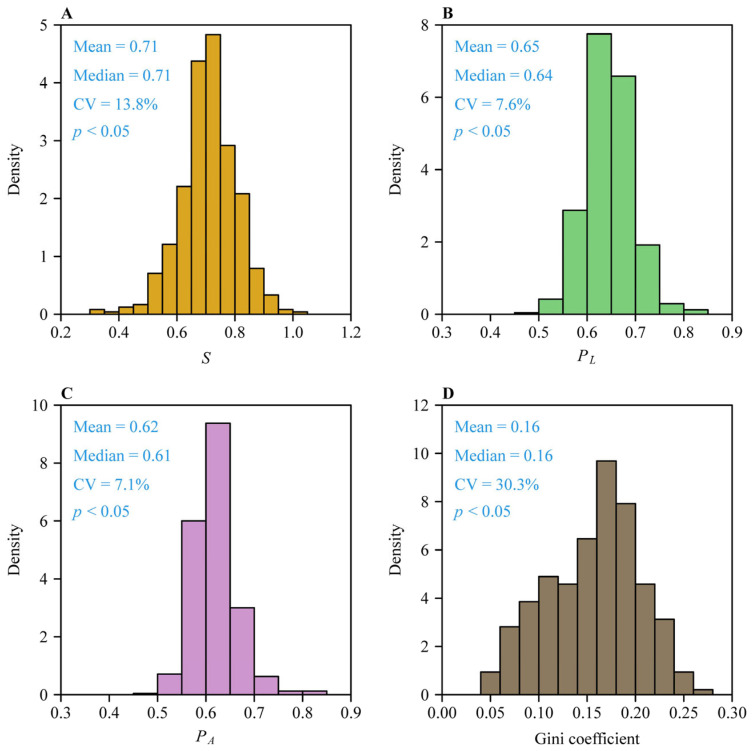
Distributions of three asymmetry measures (*S*, *P_L_*, and *P_A_*) for the Lorenz curves (**A**–**C**), and the Gini coefficients (**D**) for the 480 *S. chinensis* shoots. “Mean” and “Median” represent the mean and median, respectively; CV is the coefficient of variation; and *p* is the probability that the data are consistent with the null hypothesis of a normal distribution.

**Figure 5:**
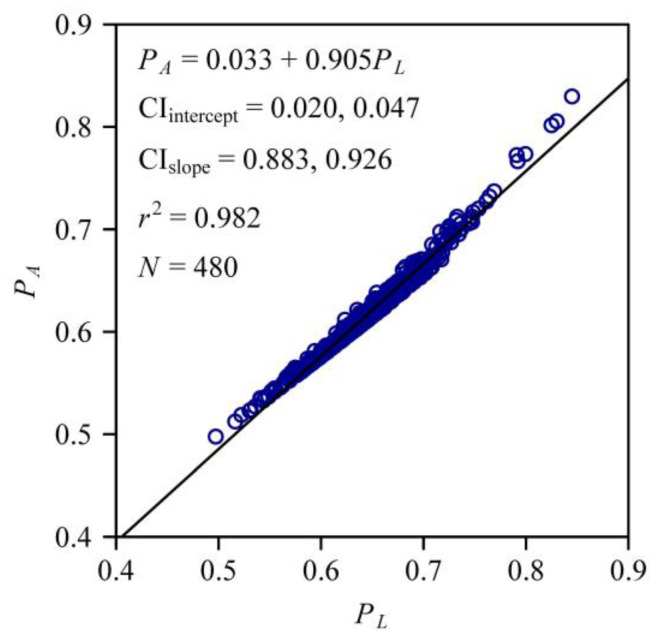
Linear fit to the two asymmetry measures (*P_A_* versus *P_L_*). The open circles represent the observations; CI_intercept_ represents the 95% confidence interval of the intercept; CI_slope_ represents the 95% confidence interval of the slope; *r*^2^ is the coefficient of determination of the linear fitting; and *N* is the sample size, i.e., the total number of shoots.

The performance equation (i.e., Equation (2)) well fitted the observations, with most shoots (98.1%, i.e., 471 out of the 480 shoots) having < 0.05 RMSE_adj_ values (Figure 2). Figure 3 exhibits an example of data fitting for a representative shoot (the 223rd shoot). The first Lorenz asymmetry coefficients (*S*, Equation (5)) ranged from 0.310 to 1.006, with the mean ± standard error 0.710 ± 0.098 (Figure 4A). The second Lorenz asymmetry coefficients (*P_L_*, Equation (6)) ranged from 0.497 to 0.845, with the mean ± standard error 0.646 ± 0.049 (Figure 4B). The third Lorenz asymmetry coefficients (*P_A_*, Equation (7)) ranged from 0.498 to 0.830, with the mean ± standard error 0.617 ± 0.044 (Figure 4C). The consistent alignment among *S*, *P_L_*, and *P_A_* metrics demonstrates that abundant larger leaves disproportionately contribute to the inequality in leaf area distribution across the 479 shoots. The Gini coefficients ranged from 0.046 to 0.272, with the mean ± standard error 0.156 ± 0.047 (Figure 4D), indicating large variation in the inequality of leaf area distributions among the 480 shoots. There was a robust linear relationship between *P_A_* and *P_L_* (*r*^2^ = 0.982; Figure 5). The estimates of model parameters, three Lorenz asymmetry measures (i.e., *S*, *P_L_*, and *P_A_*), and Gini coefficients for the 480 *S*. *chinensis* shoots are available in Table S1.

## 4. Discussion

This study re-examined the Lorenz asymmetry coefficient (*S*) through the lens of rotated and right-shifted Lorenz curves (RRLCs), proposing two novel measures (*P_L_* and *P_A_*) to quantify asymmetry in leaf area distributions of *S*. *chinensis*. By analyzing 480 shoots, we showed that 99.8% of RRLCs were left-skewed (*S* < 1 and *P_L_* or *P_A_* > 0.5), indicating a dominant role of larger leaves in driving inequality. These findings not only validate the utility of the performance equation in modeling RRLCs but also highlight the limitations of the Gini coefficient as a summary statistic of the Lorenz curve. The explicit relationship between *S* and *P_L_* (*S* = 2 − 2*P_L_*), alongside the empirical link between *P_L_* and *P_A_*, provides a robust toolkit for dissecting asymmetric patterns in biological size distributions. Below, we contextualize these results within three key themes: ecological implications of asymmetric resource allocation, methodological advancements in inequality metrics, and practical applications for plant ecology and management.

### 4.1. Ecological Implications of Asymmetric Leaf Area Distributions and Resource Allocation Strategies

The finding that most RRLCs were left-skewed (*S* < 1 and *P_L_* or *P_A_* > 0.5) highlights the predominant contribution of larger leaves to the inequality of leaf area distributions of *S*. *chinensis* shoots. This asymmetry is consistent with ecological strategies for optimizing light capture in heterogeneous environments. The Lorenz asymmetry coefficient (*S*), originally proposed by Damgaard and Weiner [19], quantifies the contribution of large-size individuals to the inequality of size distributions, a metric now complemented by two novel measures, *P_L_* and *P_A_*, for the RRLC. In shaded understories or lower canopy layers, plants often develop larger, thinner leaves to maximize light interception [4,5,6]. The observed left-skewed RRLCs may reflect adaptive basipetal gradients in leaf area, where older basal leaves expand to prolong photosynthetic activity under diminishing light availability, while younger apical leaves prioritize structural efficiency [10]. Such patterns suggest that bamboo shoots balance carbon investment across leaf ages to mitigate light limitation, a strategy critical for understory survival. Future studies could integrate light microclimate data (e.g., canopy openness) with leaf traits (e.g., specific leaf area, chlorophyll content) to test whether left-skewed distributions correlate with shade adaptation or nutrient reallocation dynamics.

### 4.2. Methodological Advancements: From the Gini Coefficient to Multidimensional Asymmetry Metrics

While the Gini coefficient provides a good overall measure of inequality, it lacks sensitivity to the asymmetry of Lorenz curves [19]. The Lorenz asymmetry coefficient *S* addressed this limitation by incorporating the contribution of large-size individuals to the Gini coefficient [19]. Building on this framework, our study introduces *P_L_* and *P_A_*, which leverage geometric properties of the RRLC to quantify its asymmetry through distinct approaches: *P_L_* reflects the positional shift of the RRLC’s maximum value point, while *P_A_* integrates the area ratio between the left and right parts of the performance curve intersecting with the *x*-axis. These metrics enhance the resolution of the analysis of inequality by disentangling the roles of outlier leaves (e.g., dominant apical leaves) versus cumulative small-leaf effects. The performance equation (Equation (2)), with parameters *K*_1_ and *K*_2_ governing curvature asymmetry, offers a flexible tool for modeling both discrete and continuous distributions. Notably, the strong relationship between *P_A_* and *P_L_* (*r*^2^ = 0.982) suggests redundancy in some applications but underscores their complementary utility in capturing different facets of asymmetry. Future work could extend this framework to other plant traits (e.g., seed mass and root biomass) and explore linkages to functional trade-offs, such as growth-defense balances [12].

### 4.3. Practical Applications and Future Directions: Bridging Theory and Management

The three asymmetry measures (*S*, *P_L_*, and *P_A_*) hold promise for ecological monitoring and management. For instance, in bamboo cultivation, shifts in *P_L_* values could indicate stress responses, e.g., nutrient limitation might amplify left-skewed distributions as plants prioritize fewer large leaves for light foraging. Conversely, right-skewed patterns (*S* > 1 or *P_L_* and *P_A_* < 0.5) might signal intense intraspecific competition, favoring smaller leaves to reduce self-shading. While the performance equation exhibited robust fits (RMSE_adj_ < 0.05 for 98.1% of the 480 shoots), its primary value lies in enabling precise parameterization of RRLCs rather than directly diagnosing anomalies. Integrating these metrics with remote sensing (e.g., UAV-based multispectral imaging) could scale analyses to canopy levels, facilitating real-time assessments of leaf distribution health. However, the current study’s focus on a single species in a uniform habitat limits our ability to generalize. Future research should validate these methods across diverse taxa (e.g., broadleaf trees and grasses) and environments (e.g., tropical vs. temperate ecosystems). Additionally, linking asymmetry metrics to ecosystem functions, such as whether left-skewed distributions enhance carbon sequestration via dominant large leaves, could deepen our understanding of plant adaptation strategies under global change.

## 5. Conclusions

The Lorenz curve was originally developed to plot the cumulative percentage of household income (*y*′) against the cumulative percentage of number of households (*x*′) to reflect the inequality of income distributions. It has also been used to describe the inequality of biological size distributions. When the Lorenz curve is rotated counterclockwise by 135° and then shifted to the right by $\sqrt{2}$ (denoted as the rotated and right-shifted Lorenz curve, RRLC), it is a skewed or symmetrical bell-shaped curve that can be well described by a nonlinear equation (referred to as the performance equation), which has two intersections, (0, 0) and ($\sqrt{2}$, 0), with the *x*-axis. Double the area of the region formed by the performance curve and the *x*-axis equals the Gini coefficient, quantifying the overall inequality. Because the performance curve might be left-skewed, right-skewed, or symmetrical, which correspond to three size distribution patterns: (i) abundant large individuals contribute most to the inequality of size distributions, (ii) a few large individuals contribute most, and (iii) small-size and large-size individuals contribute equally. However, the Gini coefficient itself cannot reflect these three patterns exhibited by the Lorenz curve. Damgaard and Weiner [19] proposed an indicator for measuring the asymmetry of the Lorenz curve named the Lorenz asymmetry coefficient (*S*), which was defined as $x_{c}^{\prime }+y_{c}^{\prime }$, where $x_{c}^{\prime }$ and $y_{c}^{\prime }$ are the horizontal and vertical coordinates of the point at which the tangent of the Lorenz curve is a 45° straight line. The cases of *S* < 1, *S* > 1, and *S* = 1 exactly correspond to the above three size distribution patterns. In the present study, we provided another expression of the Lorenz asymmetry coefficient based on the RRLC, which is defined as the proportion of the line segment from (0, 0) to the point on the *x*-axis associated with the maximum value on the RRLC to $\sqrt{2}$, denoted as *P_L_*. It provides a more intuitive measure that has an explicit mathematical relationship with *S*, that is, *S* = 2 − 2*P_L_*. In addition, a third asymmetry measure for the RRLC was proposed, which is defined as the area of the region formed by the left part of the RRLC and the *x*-axis to the area of the region formed by the whole RRLC and the *x*-axis, denoted as *P_A_*. These three measures can reflect the contribution of the largest individuals to the overall inequality. Future studies should validate the prevalence of left-skewed leaf area distributions across plant species with contrasting ecological strategies (e.g., shade-tolerant vs. pioneer species), particularly under varying light and nutrient regimes. Additionally, investigating linkages between whole-plant morphological traits (e.g., aboveground biomass, height, and crown architecture) and the proposed asymmetry metrics (*P_L_* and *P_A_*) could elucidate how interspecific and intraspecific competition shapes leaf functional trait allocation, potentially through alterations in branching patterns, vertical growth, and canopy expansion, to optimize resource acquisition in heterogeneous environments.

## Data Availability

The raw data of leaf size measures are available in the online Supplementary Tables S1 and S3 in ref. [31].

## Supplementary Materials

The following supporting information can be downloaded at https://www.mdpi.com/article/10.3390/plants14091345/s1: Table S1: Estimated parameters, goodness of fit, and measures for the Lorenz curve’s asymmetry.

## Appendix A. Relationship Between Two Asymmetry Measures

It is apparent that the maximum value point $\left(x_{c},\;y_{c}\right)$ in the *x–y* plane is obtained by rotating $\left(x_{c}^{\prime },y_{c}^{\prime }\right)$ counterclockwise by 3/4π and shifting it to the right by $\sqrt{2}$. Thus, the following is obtained:

(A1) $$\left\{\begin{array}{c} x_{c}^{\prime }=\left(x_{c}-\sqrt{2}\right)\cos\frac{3}{4}\pi +y_{c}\sin\frac{3}{4}\pi \\ y_{c}^{\prime }=-\left(x_{c}-\sqrt{2}\right)\sin\frac{3}{4}\pi +y_{c}\cos\frac{3}{4}\pi \end{array}\right..$$

Because $\sin\frac{3}{4}\pi =\frac{\sqrt{2}}{2}$ and $\cos\frac{3}{4}\pi =-\frac{\sqrt{2}}{2}$ we obtain the following:

(A2) $$S=x_{c}^{\prime }+y_{c}^{\prime }=2-\sqrt{2}x_{c}.$$

According to the definition of *P_L_* (Equation (6)), we obtain the following:

(A3) $$S=2-2P_{L}$$

## References

[1] Evans J.R. (1999). Leaf anatomy enables more equal access to light and CO_2_ between chloroplasts. New Phytol..

[2] Taiz L., Møller I.M., Murphy A., Zeiger E. (2022). Plant Physiology and Development.

[3] Küppers M. (1989). Ecological significance of above-ground architectural patterns in woody plants: A question of cost-benefit relationships. Trends Ecol. Evol..

[4] de Casas R.R., Vargas P., Pérez-Corona E., Manrique E., García-Verdugo C., Balaguer L. (2011). Sun and shade leaves of *Olea europaea* respond differently to plant size, light availability and genetic variation. Funct. Ecol..

[5] Niinemets Ü. (2001). Global-scale climatic controls of leaf dry mass per area, density, and thickness in trees and shrubs. Ecology.

[6] Terashima I., Hanba Y.T., Tazoe Y., Vyas P., Yano S. (2006). Irradiance and phenotype: Comparative eco-development of sun and shade leaves in relation to photosynthetic CO_2_ diffusion. J. Exp. Bot..

[7] Poorter H., Niinemets Ü., Poorter L., Wright I.J., Villar R. (2009). Causes and consequences of variation in leaf mass per area (LMA): A meta-analysis. New Phytol..

[8] Dörken V.M., Lepetit B. (2018). Morpho-anatomical and physiological differences between sun and shade leaves in *Abies alba* Mill. (Pinaceae, Coniferales): A combined approach. Plant Cell Environ..

[9] Hirose T., Werger M.J.A. (1987). Maximizing daily canopy photosynthesis with respect to the leaf nitrogen allocation pattern in the canopy. Oecologia.

[10] Kikuzawa K. (2003). Phenological and morphological adaptations to the light environment in two woody and two herbaceous plant species. Funct. Ecol..

[11] Lian M., Shi P., Zhang L., Yao W., Gielis J., Niklas K.J. (2023). A generalized performance equation and its application in measuring the Gini index of leaf size inequality. Trees Struct. Funct..

[12] Givnish T.J. (1988). Adaptation to sun and shade: A whole-plant perspective. Aust. J. Plant Physiol..

[13] Lorenz M.O. (1905). Methods of measuring the concentration of wealth. Am. Statist. Assoc..

[14] Gastwirth J.L. (1971). A general definition of the Lorenz curve. Econometrica.

[15] Sarabia J.-M. (1997). A hierarchy of Lorenz curves based on the generalized Tukey’s lambda distribution. Econom. Rev..

[16] Gini C. (1912). Variability and Mutability: Contribution to the Study of Distributions and Statistical Relationships.

[17] Weiner J., Solbrig O.T. (1984). The meaning and measurement of size hierarchies in plant populations. Oecologia.

[18] Shi P., Deng L., Niklas K.J. (2024). Rotated Lorenz curves of biological size distributions follow two performance equations. Symmetry.

[19] Damgaard C., Weiner J. (2000). Describing inequality in plant size or fecundity. Ecology.

[20] Huey R.B., Stevenson R.D. (1979). Integrating thermal physiology and ecology of ectotherms: A discussion of approaches. Amer. Zool..

[21] Ratkowsky D.A. (1983). Nonlinear Regression Modeling.

[22] Ratkowsky D.A. (1990). Handbook of Nonlinear Regression Models.

[23] Bates D.M., Watts D.G. (1988). Nonlinear Regression Analysis and Its Applications.

[24] Lian M., Chen L., Hui C., Shi P. (2024). On the relationship between the Gini coefficient and skewness. Ecol. Evol..

[25] Shi P., Ratkowsky D., Li Y., Zhang L., Lin S., Gielis J. (2018). General leaf-area geometric formula exists for plants—Evidence from the simplified Gielis equation. Forests.

[26] Su J., Niklas K.J., Huang W., Yu X., Yang Y., Shi P. (2019). Lamina shape does not correlate with lamina surface area: An analysis based on the simplified Gielis equation. Glob. Ecol. Conserv..

[27] Shi P., Gielis J., Quinn B.K., Niklas K.J., Ratkowsky D.A., Schrader J., Ruan H., Wang L., Niinemets Ü. (2022). ‘biogeom’: An R package for simulating and fitting natural shapes. Ann. N. Y. Acad. Sci..

[28] R Core Team (2022). R: A Language and Environment for Statistical Computing.

[29] Montgomery E.G. (1911). Correlation Studies in Corn.

[30] Schrader J., Shi P., Royer D.L., Peppe D.J., Gallagher R.V., Li Y., Wang R., Wright I.J. (2021). Leaf size estimation based on leaf length, width and shape. Ann. Bot..

[31] Wang C., Heng Y., Xu Q., Zhou Y., Sun X., Wang Y., Yao W., Lian M., Li Q., Zhang L. (2024). Scaling relationships between the total number of leaves and the total leaf area per culm of two dwarf bamboo species. Ecol. Evol..

[32] Niklas K.J. (1994). Plant Allometry: The Scaling of Form and Process.

[33] Shi P., Ge F., Sun Y., Chen C. (2011). A simple model for describing the effect of temperature on insect developmental rate. J. Asia-Pacific Entomol..

[34] Byrd R.H., Lu P., Nocedal J., Zhu C. (1995). A limited memory algorithm for bound constrained optimization. SIAM J. Sci. Comput..

[35] Shi P., Chen L., Quinn B.K., Yu K., Miao Q., Guo X., Lian M., Gielis J., Niklas K.J. (2023). A simple way to calculate the volume and surface area of avian eggs. Ann. N. Y. Acad. Sci..

